# External Validation of the Prognostic Value of an Immune-Associated Gene Panel for Clear Cell Renal Cell Carcinomas

**DOI:** 10.3389/fcell.2021.794840

**Published:** 2021-12-23

**Authors:** Zhiwen Xie, Lei Wu, Shan Hua, Yongqing Zhang, Fei Shi, Min Chen, Sheng Zhao, Zhihong Liu, Meng Liu, Juntao Jiang

**Affiliations:** ^1^ Department of Urology, Shanghai General Hospital, Shanghai Jiao Tong University School of Medicine, Shanghai, China; ^2^ Department of Urology, Shanghai General Hospital of Nanjing Medical University, Shanghai, China; ^3^ Department of General Surgery, Shanghai Ninth People’s Hospital Affiliated to Shanghai Jiao Tong University School of Medicine, Shanghai, China; ^4^ Department of Urology, Sir Run Run Shaw Hospital, Zhejiang University School of Medicine, Hangzhou, China; ^5^ Department of Urology, The Fifth People’s Hospital of Zunyi, Guizhou, China

**Keywords:** clear cell renal cell carcinomas, immunotherapy, immune gene, prognosis panel, nomogram

## Abstract

Clear cell renal cell carcinomas (ccRCCs) are highly immune infiltrates, and many of them respond to immunotherapy with checkpoint inhibitors including anti-PD-L1 or anti-PD1 agents. However, the effect of immune genes on clinical outcomes in ccRCCs has not been fully studied. Here, we show in this study that an immune-associated gene panel has a prognostic value for clear cell renal cell carcinomas. We performed single-sample gene set enrichment analysis (ssGSEA) and cell type identification by estimating subsets of RNA transcripts (CIBERSORT) algorithms on patient-matched normal renal and RCC tissues to characterize two immunophenotypes and immunological characteristic subpopulations. Furthermore, LASSO Cox regression was applied to develop a novel prognosis-associated model for ccRCC patients based on an immune-gene panel. The results were verified by the Gene Expression Omnibus (GEO) dataset and coordinated with the clinicopathological characteristics of ccRCCs, along with genomic signatures. Finally, based on the above perspectives, we generated a nomogram with a high prognostic efficiency for ccRCC patients. Overall, this study offers a unique perspective that can contribute to improving the accuracy of prognosis prediction and treatment with immunotherapy.

## Introduction

Renal cell carcinoma (RCC) is a common tumor of the urinary system and accounts for approximately 3% of all adult cancers (Zarrabi et al., 2017), with an annual increase of more than 0.40 million newly diagnosed and 0.18 million deaths (Capitanio et al., 2019). RCC exists twice as often in men as in women, and the highest prevalence of RCC is often observed in patients in their sixties (Mulders et al., 1997). In addition, the incidence of RCC is higher in developed countries than in developing countries. The highest levels were observed in Europe, North America, and Australia, with the lowest levels being found in Africa, India, and China (Hsieh et al., 2017). Most patients are frequently diagnosed with RCC after presenting with typical symptoms, such as hematuria, flank pain, and palpable abdominal mass, thereby missing the optimal treatment time (Chevrier et al., 2017). Based on histological and cytogenetic characteristics, 80% of RCC is subclassified as clear cell renal cell carcinoma (ccRCC), which originates from nephron epithelial cells and has the highest mortality rate of all urinary tumors (McDougal et al., 2015). The prognosis of RCC remains poor. Especially for patients with advanced and metastatic disease, the 5-year survival rate is only 23% after diagnosis (Ljungberg et al., 2015). Presently, surgery is the main treatment for ccRCC, but this is correlated with a high incidence of recurrence and metastasis (Sorokin et al., 2017). Furthermore, ccRCCs are radioresistant (Bielecka et al., 2014), and more than 80% are chemoresistant (Clerici and Boletta, 2020), with agents producing a positive response only in a minority of patients. Since ccRCCs are presumed to be immunogenic (Xu et al., 2020), immunotherapy has been widely employed and shows promising clinical effects in the treatment of renal cancer. However, the rapid development of resistance during applied targeted therapy has become an increasingly challenging issue (Miao et al., 2018; Braun et al., 2020).

Currently, the clinical prognosis of ccRCC is predicted by multiple factors, including clinical, anatomical, molecular, and histological features, such as DNA methylation genes, inflammasome-related signatures, Notch signaling pathways, and RNA-binding proteins (Zhou et al., 2020). However, none of these approaches have yet improved the current prognostic systems. Nishida (Nishida et al., 2020) reported that immune genes were associated with clinical outcomes. Basically, the lack of reliable and stable prognostic markers has been a great obstacle, but immune-associated genes may provide a novel insight into this field (Qi et al., 2020).

In our study, we focused our efforts on illustrating the interaction between immunity and tumors and the predictive value of immune genes for ccRCCs. Consequently, by using machine learning–based approaches, we designed an immune-associated gene panel to predict the prognosis status of ccRCC patients and confirmed its stability and reliability in an independent dataset (Lin et al., 2020). Thus, it is hoped that this study will lead to a better understanding of a more effective diagnostic and therapeutic indicator for ccRCC patients.

## Materials and Methods

### Data Collection and Collation

We acquired transcriptomic data from public databases with clinicopathological features. TCGA-KIRC (kidney renal clear cell carcinoma) and GSE29609 (http://www.ncbi.nlm.nih.gov/geo/query/acc.cgi?acc=GSE29609) datasets were downloaded in our study (Giraldo et al., 2017). Immune-related genes were obtained from the ImmPort database (https://www.immport), and transcription factor–related genes were downloaded from the CISTROME project (http://www.cistrome.org/) (Meng et al., 2021). In addition, the tumor mutation burden (TMB) was calculated according to the number of gene mutations in each tumor sample (Li et al., 2020).

### Evaluation of the Tumor Microenvironment (TME)

To calculate the immune score, stromal score, and estimate score, the ESTIMATE (Estimation of STromal and Immune cells in MAlignant Tumor tissues using Expression data) algorithm was applied to the TCGA-KIRC dataset with the “estimate” R package (version 1.0.13) (Yoshihara et al., 2013). The CIBERSORT (cell type identification by estimating relative subsets of RNA transcripts) algorithm was performed to evaluate the relative proportions of 22 tumor-infiltrating cells for each ccRCC patients (Newman et al., 2015).

### Enrichment and Hierarchical Clustering Analysis

Depending on 29 immune gene sets (Supplementary Table S3), the ssGSEA algorithm was conducted to systematically assess the immunological features with the “GSVA” (version 1.38.2), “GSEABase” (version 1.52.1), and “limma” (version 3.46.0) R packages. Then, the ssGSEA score xi for each ccRCC patient was transformed into xi’ by the equation xi’= (xi − x_min_)/(x_max_ − x_min_). In addition, hierarchical clustering analysis was performed to identify the subtypes of ccRCC patients with Euclidean distance and Ward’s linkage method (Yi et al., 2020). The t-SNE (T-distribution stochastic neighbor embedding) algorithm was employed to demonstrate the accuracy and discrimination of the subtypes of ccRCC patients with the “Rtsne” (version 0.15) package (Kobak and Berens, 2019).

### The Differential and Prognostic Immune Gene Analysis

Differentially expressed genes (DEGs) were isolated between the high- and low-immunity groups *via* the “limma” R package. Statistical differences were defined as |log2 fold change| > 0.58 and FDR <0.05. Then, the differentially expressed immunity genes (DEIGs) were the intersection of genes between DEGs and the immune gene dataset (Ritchie et al., 2015). Moreover, based on DEIGs, we performed univariate survival analysis with the “survival” (version 3.2-13) R package and considered prognostic immunity genes (PIGs) when *p* < 0.05.

### Functional and Network Analysis

To identify the essential signaling pathways involving DEGs, gene set enrichment analysis (GSEA, version 4.1.0) was applied to the TCGA-KIRC dataset, which demonstrated the Kyoto Encyclopedia of Genes and Genomes (KEGG) pathways that were increased in the high- and low-immunity groups, respectively. Statistical significance was defined as FDR <0.01. In addition, we obtained differentially expressed transcription factors (DETFs) by extracting the intersection of genes between DEGs and the TF dataset (Wu and Zhang, 2018).Then, correlation analysis was performed to establish the regulatory network of DETFs and PIGs by using the Pearson’s correlation coefficient. Moreover, based on the STRING (string-db.org/) database, we conducted protein–protein interaction (PPI) analysis (Wang et al., 2020).

### Development and Validation of a Prognostic Model

Based on PIGs, we performed LASSO-Cox regression analysis to develop an immune gene–associated prognostic model (IGPM) for ccRCC patients (McEligot et al., 2020). The risk score of each patient was calculated according to the following formula: risk score = sum (the expression level of each gene × corresponding coefficient). According to the median risk score, we divided ccRCC patients into high- and low-risk groups. Then, Kaplan–Meier analysis and log rank tests were used to demonstrate the survival difference between the high‐ and low‐risk groups. To test the predictive capacity of the IGPM‐based risk model, the area under the time-dependent receiver operating curve (ROC) was calculated to predict the 1-year, 3-year, and 5-year survival rates of ccRCC patients. We conducted Kaplan–Meier log-rank tests and ROC analysis to demonstrate the survival difference and accuracy of the risk signature *via* the “timeROC” (version 0.4), “rms” (version 6.2-0), “survival” (version 3.2-13), and “survminer” (version 0.4.9) R packages. We used Pearson’s correlation or the Spearman correlation to confirm the association between the IGPM‐based risk model and clinicopathological features, TMB, immune checkpoint molecules, and immune cell infiltration *via* the “corrplot” (version 0.84) R package (Pesenti et al., 2019). We considered there to be a significant correlation when *p* < 0.05. Finally, based on the risk signature and clinicopathological features, we performed univariate and multivariate Cox regression analyses to demonstrate the independence of the risk signature. We then utilized the above characteristics to construct a nomogram and conducted ROC and calibration curve analysis (Iasonos et al., 2008) to confirm the possibility of its clinical application for the nomogram.

### RNA Extraction and Real-Time Quantitative Polymerase Chain Reaction

Total RNA were extracted from the ccRCC cell-Line 769-P and normal cell line HK-2 using TRIzol reagent (Novabio, China). Cell lines were obtained from the Chinese Academy of Sciences Committee on Type Culture Collection Cell Bank (Shanghai, China). RNA (1 mg) was reverse-transcribed to complementary DNA (cDNA) by using a PrimeScript RT kit (Novabio, China). According to the manufacturer’s manual, real-time quantitative polymerase chain reaction (RT-qPCR) was performed with gene-specific primers to determine the relative expression of genes of interest using SYBR green and was analyzed by using an ABI 7500 Real-Time PCR system (Applied Biosystems). All experiments were performed to obtain three independent measures.

## Results

### Two Immune Subtypes of ccRCC Were Identified by Immunogenomic Profiling

**FIGURE 1:**
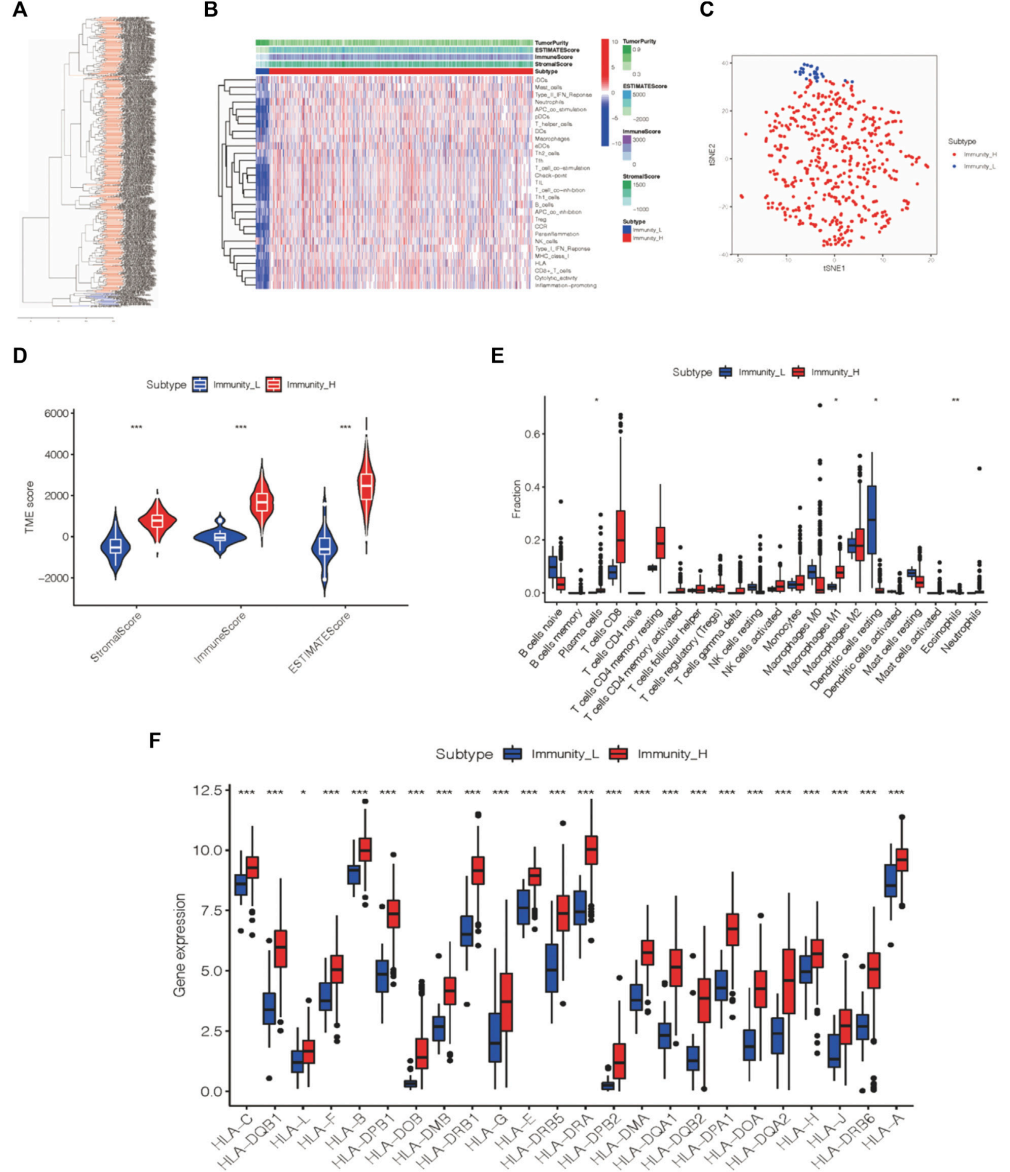
Two immune subtypes of ccRCC were established *via* hierarchical clustering. **(A)** ssGSEA results were used to generated two distinct groups *via* hierarchical clustering. **(B)** Tumor microenvironment characteristics were generated in the TCGA-KIRC cohort. **(C)** Immunophenotype validation *via* tSNE. **(D)** Three different immune scores of each group. **(E)** Immune cell infiltration degree of two immunogenic subtypes. **(F)** HLA-associated gene level of each of the two groups. *p < 0.05, **p < 0.01, ***p < 0.001.

Together with 29 immune gene sets, 539 tumor patient samples from the TCGA-KIRC cohort were comprehensively assessed by the ssGSEA algorithm to evaluate the immunological features (Xu et al., 2019a). Two immune subtypes, Immunity_High (Immunity_H) and Immunity_Low (Immunity_L), were divided in consonance with the ssGSEA scores and hierarchical clustering analysis (Figures 1A,B). The results of the tSNE algorithm confirmed the above classification (Figure 1C).

The ESTIMATE algorithm was applied to analyze the tumor microenvironment characteristics of these two subtypes. We found that the Immunity_H group exhibited more scores for the stromal score, immune score, and estimate score (Wilcox test, *p* < 0.001) than the Immunity_L group (Figure 1D).

Likewise, the infiltration levels of plasma cells, M1 macrophages, dendritic cells, and eosinophils in the Immunity_H group also varied from those in the Immunity_L group according to the outcome of the CIBERSORT algorithm (Figure 1E). Furthermore, we analyzed the expression of HLA-related genes in each group, and the Immunity_H group expressed more HLA-related genes than the other group (Figure 1F). Thus, the two distinct immune groups of ccRCC patients displayed various features.

### Differentially Expressed Immune Genes and Their Prognostic Value in ccRCC Patients

Based on the abovementioned immunophenotypes, we further explored the molecular features of cross-talk between tumors and immunity and their prognostic value in ccRCC patients.

**FIGURE 2:**
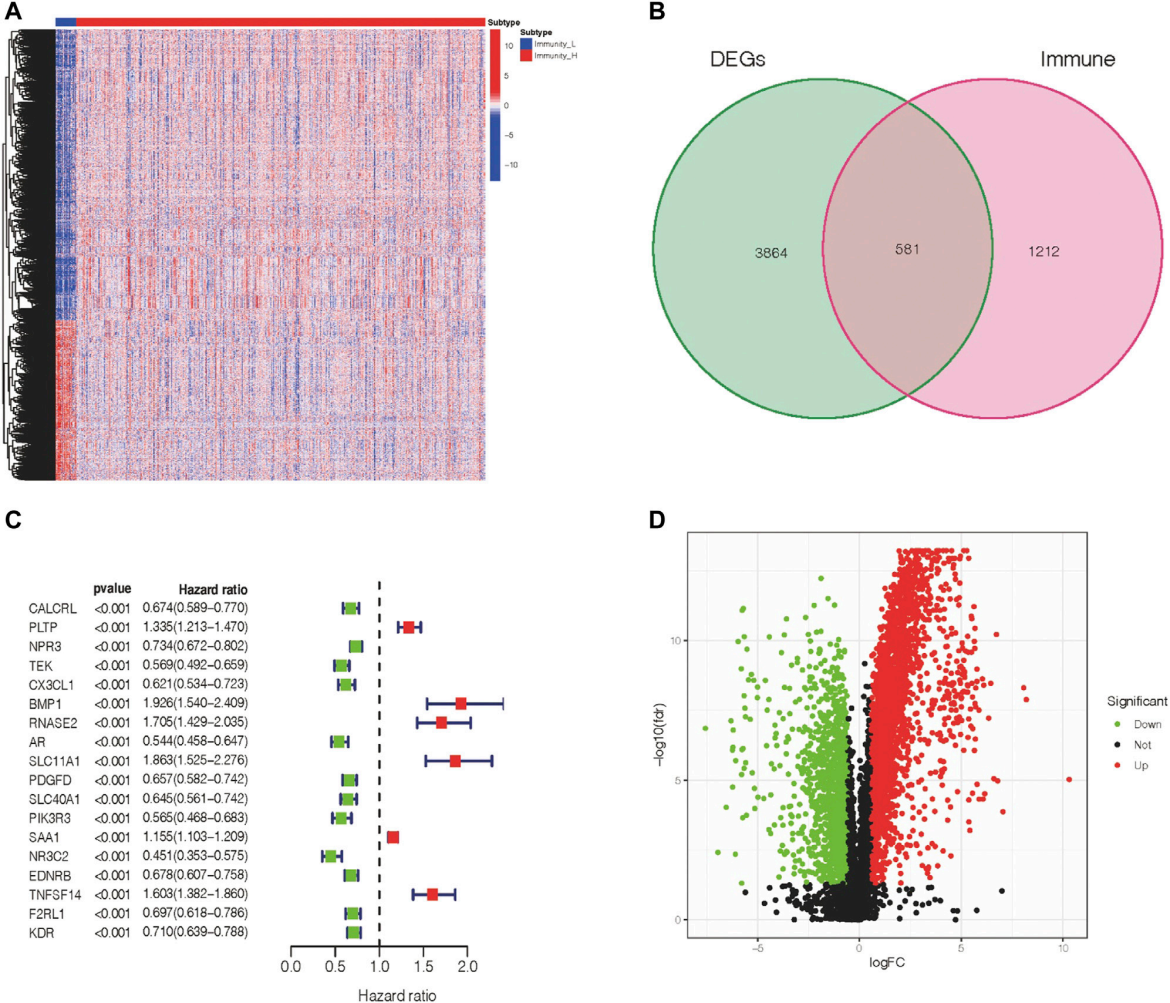
Expression of DEGs and association with the clinical outcome. **(A)** Differentially expressed genes within two immune groups. **(B)** Isolation of differentially expressed immune genes. **(C)** PIGs and their hazard ratios were displayed in the forest plot *via* univariable Cox proportional hazards analysis. **(D)** Fold change and the FDR value were displayed in the volcano plot.

We observed 4445 DEGs in the volcano map (Figure 2A). Among these DEGs, 2873 DEGs were upregulated, and 1572 DEGs were downregulated. In addition, the expression levels of these DEGs in ccRCC patients are shown in Figure 2B. Furthermore, based on the immune gene set, 581 genes were identified as DEIGs (Figure 2C). Finally, 18 PIGs were screened using a univariate Cox proportional-hazards model (Figure 2D). Of these genes in the Immunity_H subtype, 12 genes (*CALCRL, NPR3, TEK, CX3CL1, AR, PDGFD, SLC40A1, PIK3R3, NR3C2, EDNRB, F2RL1,* and *KDR*) were downregulated and six genes (*PLTP, BMP1, RNASE2, SLC11A1, SAA1,* and *TNFSF14*) were upregulated.

### Cross-Talk Between Tumors and Immunity in ccRCC Patients

**FIGURE 3:**
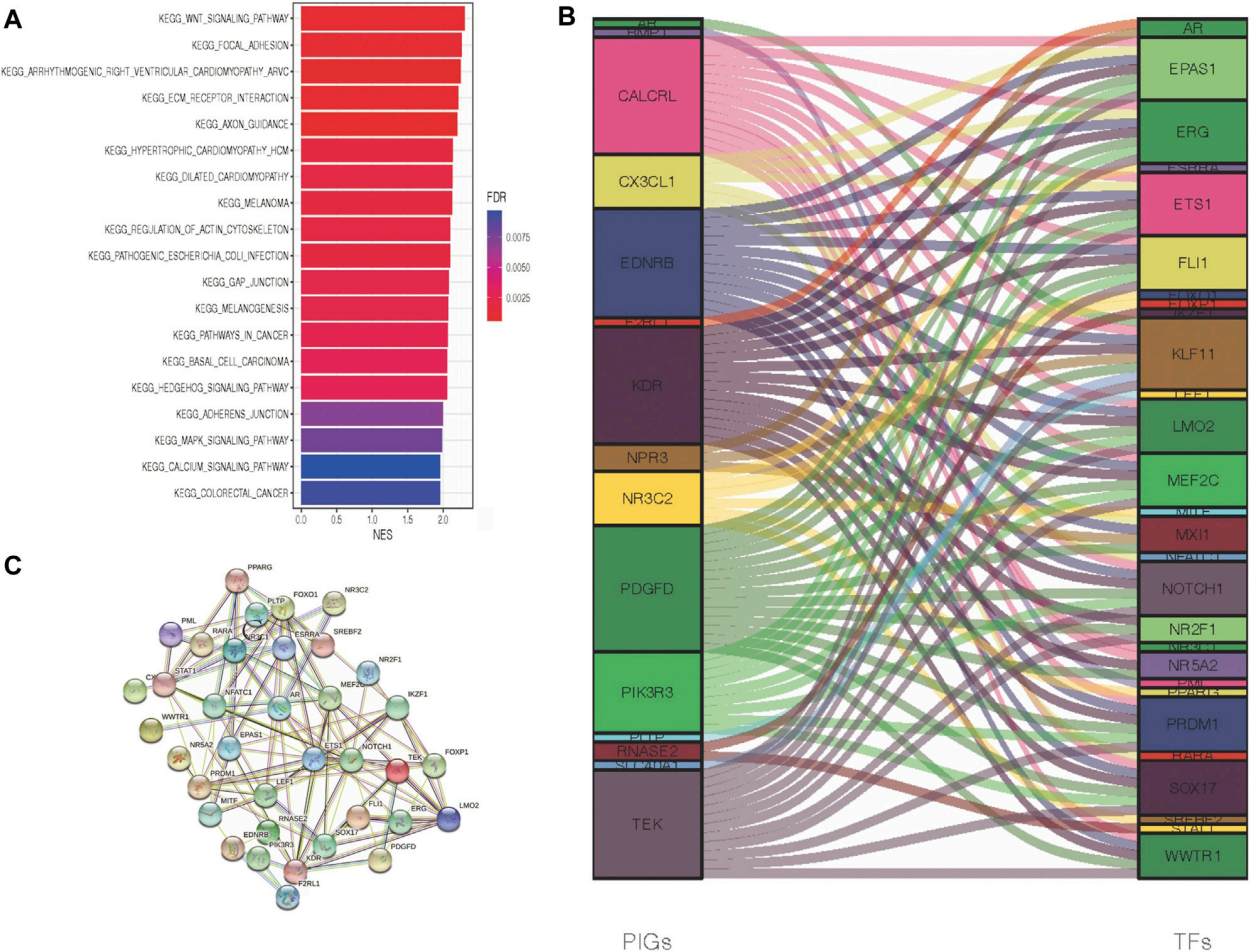
Crosstalk networks and pathway characterization between each of the two immune subtypes. **(A)** GSEA and KEGG analyses between DEGs of two immune subtypes. **(B)** TF and PIG regulatory crosstalk were displayed *via* an alluvial diagram. **(C)** PPI network between TFs and PIGs.

To obtain a comprehensive evaluation of the role that immune genes play in the biological processes of ccRCC patients, we conducted GSEA to characterize the pathways in which these DEGs were engaged (Figure 3A). According to KEGG pathway analysis, melanoma, basal cell carcinoma, colorectal cancer, and cancer-related pathways were especially enriched in the Immunity_H group, while no significant pathways were enriched in the Immunity_L group. Briefly, increased activation of the immune part in the Immunity_H group (Supplementary Table S1) was positively associated with several cancer-related pathways in Immunity_H ccRCC patient samples, and it was validated by GSEA. To further clarify the underlying mechanism between immune-associated genes and the development or prognosis of ccRCC patients, we further investigated the upstream PIGs. Then, by comparing differential expression results with the CISTROME gene set, we characterized transcription factors (TFs) that are essential for the development and metastasis of ccRCC.

In the above experiments, we generated 29 upregulated transcription factors in the Immunity_H group. Subsequently, a functional network of the TF-PIGs (Figure 3B) was constructed by correlation analysis. All these TFs of ccRCC were positively associated with their interrelated PIGs (Supplementary Table S2). Finally, a PPI (protein–protein interaction) analysis was conducted to validate the TF-PIG interplay, and the presence of a strong connection between each of them was proved (Figure 3C).

### Establishment and Validation of This Prognosis-Associated Gene Panel

**FIGURE 4:**
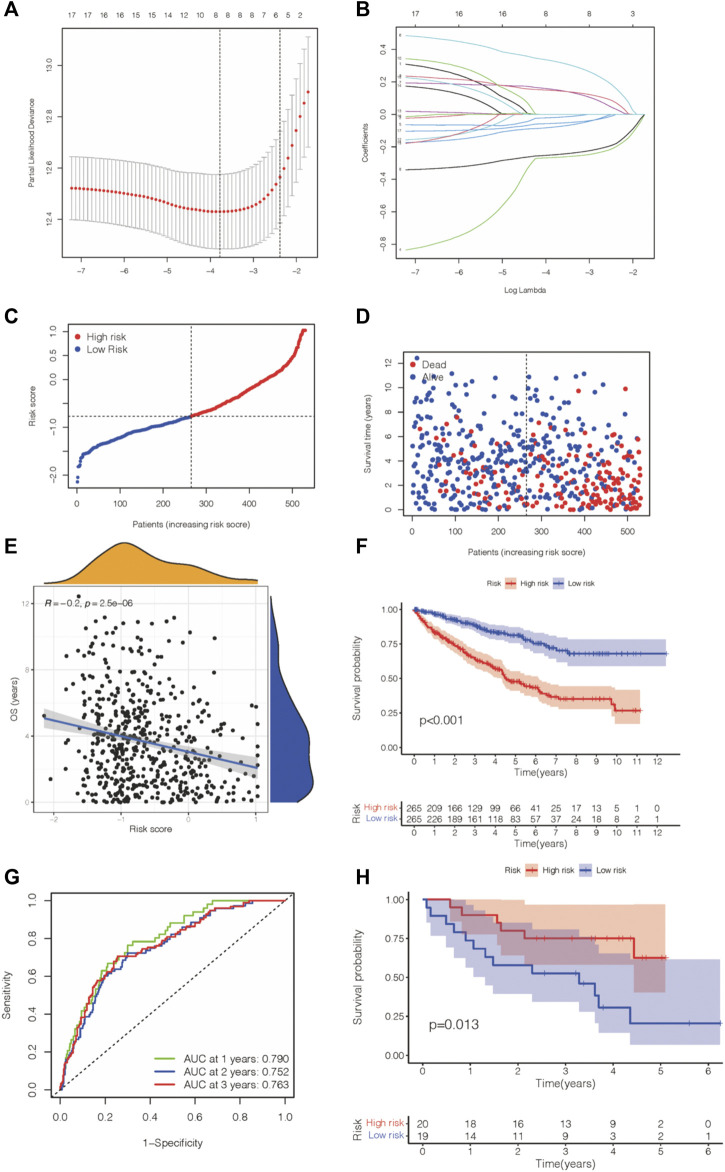
IGPM-based prognosis panel was constructed and verified. **(A**,**B)** LASSO-Cox regression analysis was applied to construct the prediction model for ccRCC prognosis. **(C**,**D)** Risk value and survival information were shown in KIRC. **(E)** Correlation analysis of the risk value and survival time in KIRC. **(F)** Kaplan–Meier survival analysis of the KIRC and gene panel. **(G)** The AUC score of the accuracy of the predictive prognosis panel for KIRC. **(H)** Kaplan–Meier survival analysis of the gene panel was conducted in GSE29609.

First, LASSO-Cox analysis was conducted to reduce the dimensions of these PIGs. Then, to construct an immune gene–associated gene panel for prognosis prediction, we employed IGPM to design an eight-parameter formula for overall survival prediction and prognosis prediction in the TCGA-KIRC database. This risk value formula was (−0.265) * expression of TEK + (−0.019) * expression of CX3CL1 + 0.325 * expression of BMP1 + 0.152 * expression of RNASE2 + (−0.248) * expression of AR + 0.146 * expression of SLC11A1+ (−0.077) * expression of SLC40A1+ (−0.055) * expression of F2RL1 (Figures 4A,B). Additionally, we determined the risk value of ccRCC patients, and then, in accordance with the average risk value, we separated this cohort into two distinct groups: high-risk and low-risk groups (Figure 4C). The survival analysis revealed that the low-risk group had a longer survival time and exhibited a lower mortality rate than the high-risk group (Figure 4D). Meanwhile, we performed a correlation analysis, and the previous outcome confirmed that a higher risk value was associated with a decreased survival time (Figure 4E), suggesting a negative relationship between the risk value and survival time in the ccRCC cohort. Kaplan–Meier survival analysis also showed that the lower-risk group in the ccRCC cohort was related to better overall survival (Figure 4F). Moreover, we drew an ROC curve based on time to verify the stability and accuracy of this panel, and the 1-year, 2-year, and 3-year AUC values of the ROC curve were 0.790, 0.752, and 0.763, respectively (Figure 4G). Then, we sought to use the GEO database to verify the prognostic value of this panel in another independent cohort. In contrast, Kaplan–Meier survival analysis suggested that the high-risk group presented a longer survival time in the GSE29609 cohort (Figure 4H).

### Association Between the Immune Gene–Associated Prognosis Panel and Immune Compositions, Immune Checkpoint, and TMB

**FIGURE 5:**
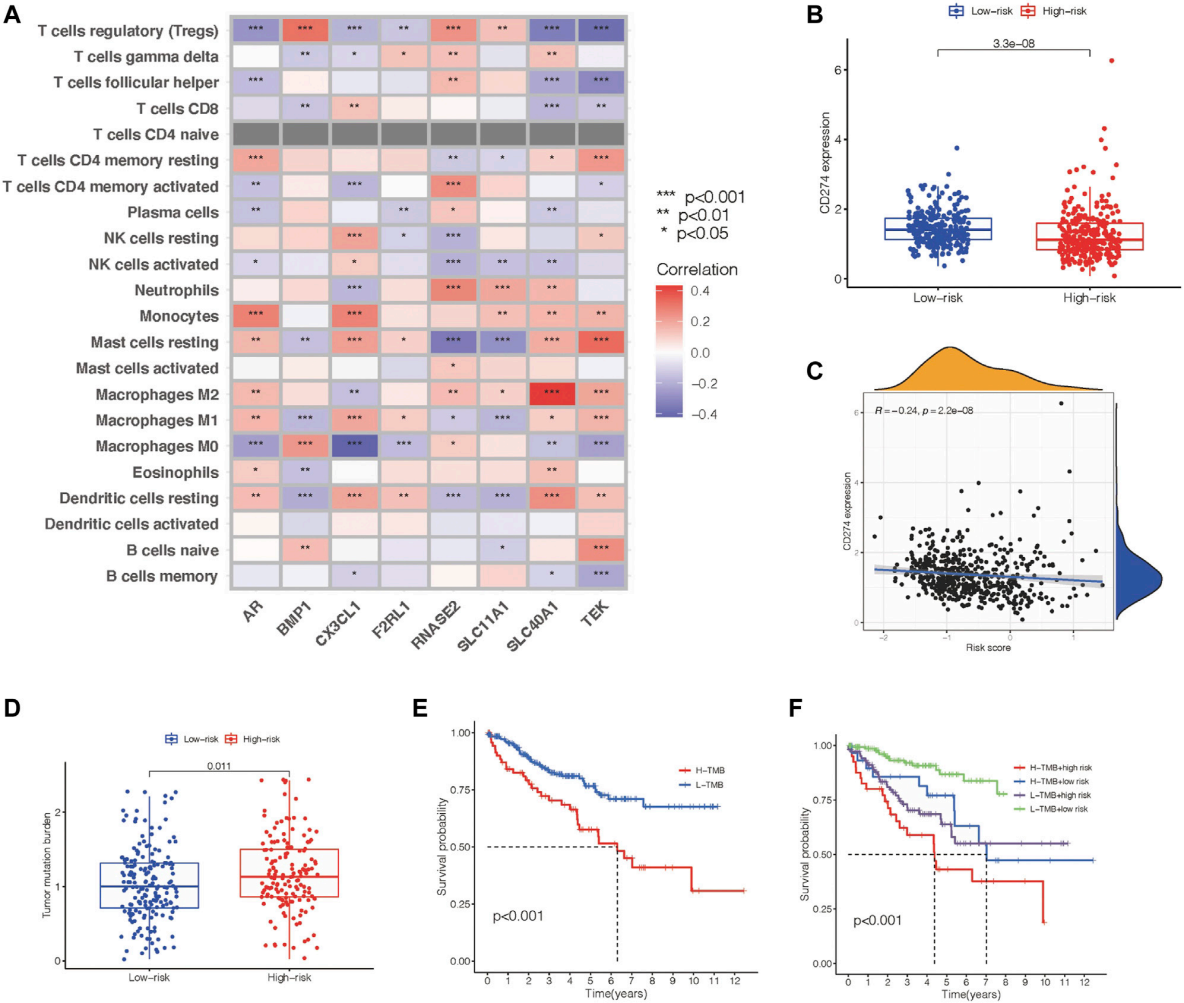
Identification of the communication between the prognosis gene panel and immune associated features. **(A)** Heatmap showing the relationship of the prognosis panel with immune compositions. **(B)** Scatter plots draw a relationship between the prognosis panel and gene expression of CD274. **(C)** Scatter plots draw a relationship between the prognosis panel and gene expression of CD274. **(D**,**E)** TMB of each group and their association with survival time. **(F)** Kaplan–Meier survival analysis of the prognosis gene panel accompanied with the TMB value.

We then checked the relevance between this predictive gene panel and several other relevant parameters, including immune compositions, immune checkpoints, clinical characteristics, and TMB. Treg cells, resting mast cells, M1macrophages, and resting dendritic cells were correlated with the eight PIGs, proving that these immune components have the capability to influence the clinical outcomes of ccRCC patients (Figure 5A). Furthermore, CD274, an immune checkpoint–associated gene, was characterized to analyze the therapeutic value of this eight-gene panel. The expression level of CD274 was negatively related to the risk value of this model in ccRCC patients, suggesting a predictive function in treatment (Figures 5B,C). As a consequence of immune checkpoints, the high-risk group patients suffer from a poorer survival time and prognosis.

Meanwhile, tumor mutation burden has the potential to predict the effect of immunotherapy for tumor patients. The TMB level was proven to be in accordance with the risk value of this prognostic model (Figure 5D). Nevertheless, Kaplan–Meier survival analysis revealed a negative relationship between TMB and overall survival (Figure 5E). This led us to add TMB to our previous model to construct a better tool to predict the prognosis of ccRCC patients. All ccRCC patients were divided into groups including high-risk patients with high TMB, high-risk patients with low TMB, low-risk patients with high TMB, and low-risk patients with low TMB. Kaplan–Meier survival analysis revealed that the overall survival time varied distinctly between each group, and the low-risk patients with low TMB has the longest overall survival time (Figure 5F).

### Establishment and Validation of a Model Combined With Clinical Characteristics

**FIGURE 6:**
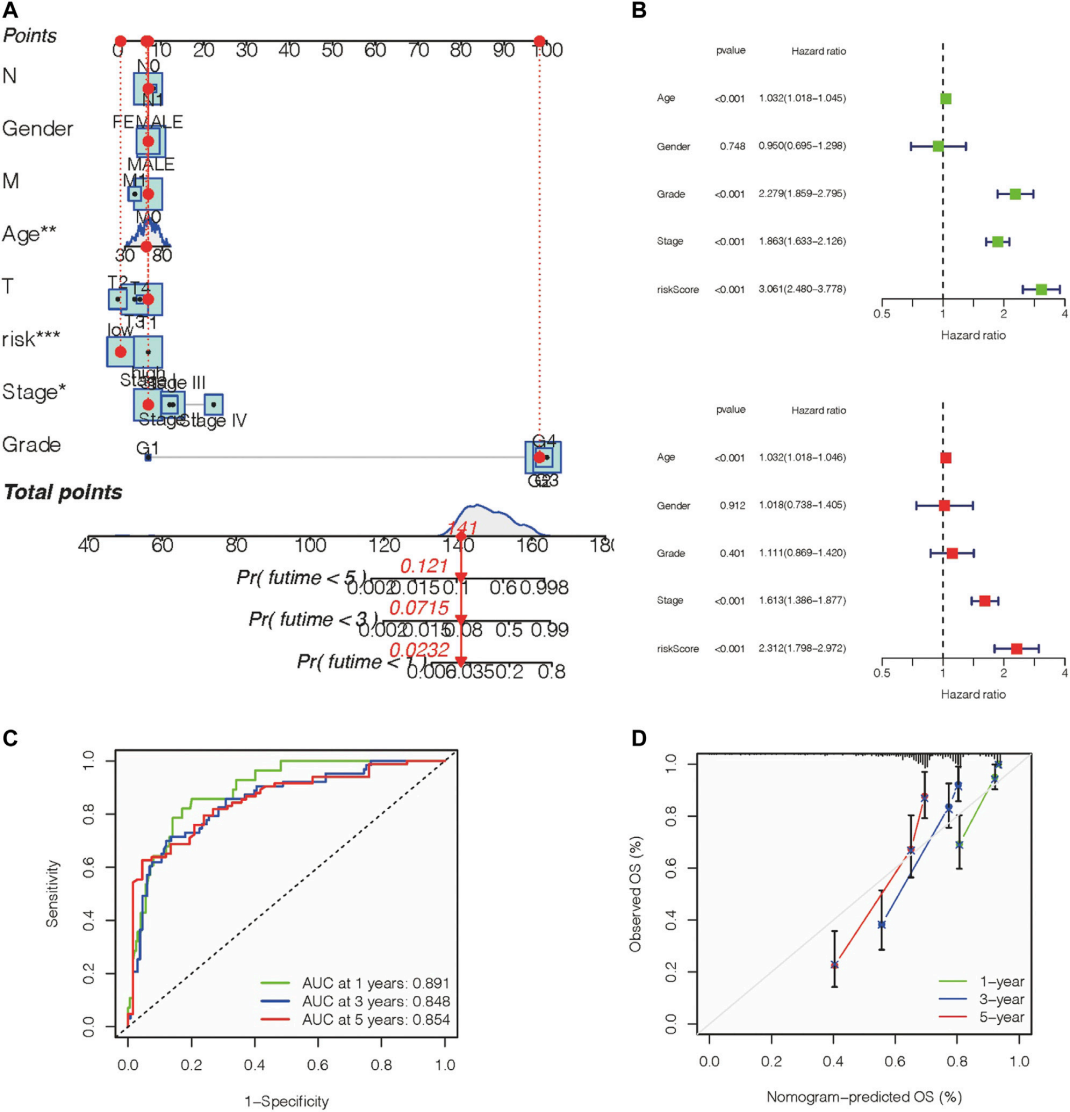
Construction of a nomogram with a predictive value for prognosis. **(A**,**B)** A nomogram was constructed based on the prognosis gene panel and other relevant features. **(C)** AUC value of the gene panel for 1-year, 3-year, 5-year prognosis prediction in the KIRC database. **(D)** Calibration plot designed for the KIRC database.

The above results demonstrated that the prognostic gene panel had a strong connection with the clinical outcome. Furthermore, we employed univariate and multivariate Cox regression analyses to investigate whether this gene panel could be used as an independent factor for prognosis prediction in ccRCC patients. When combining this risk value with several clinical features, including age, gender, TNM, and molecular characteristics, we concluded that this gene panel can be used as an independent factor for prognosis prediction, as shown in Figures 6A,B. Furthermore, a more sensitive framework was constructed by using all parameters mentioned before, and the AUC value was obtained in the ROC curve, indicating a better prognostic value (Figure 6C). In the end, a nomogram was established to better predict the prognosis outcome (Figure 6D).

### Prognostic Gene Panel Verified by RT-qPCR

**FIGURE 7:**
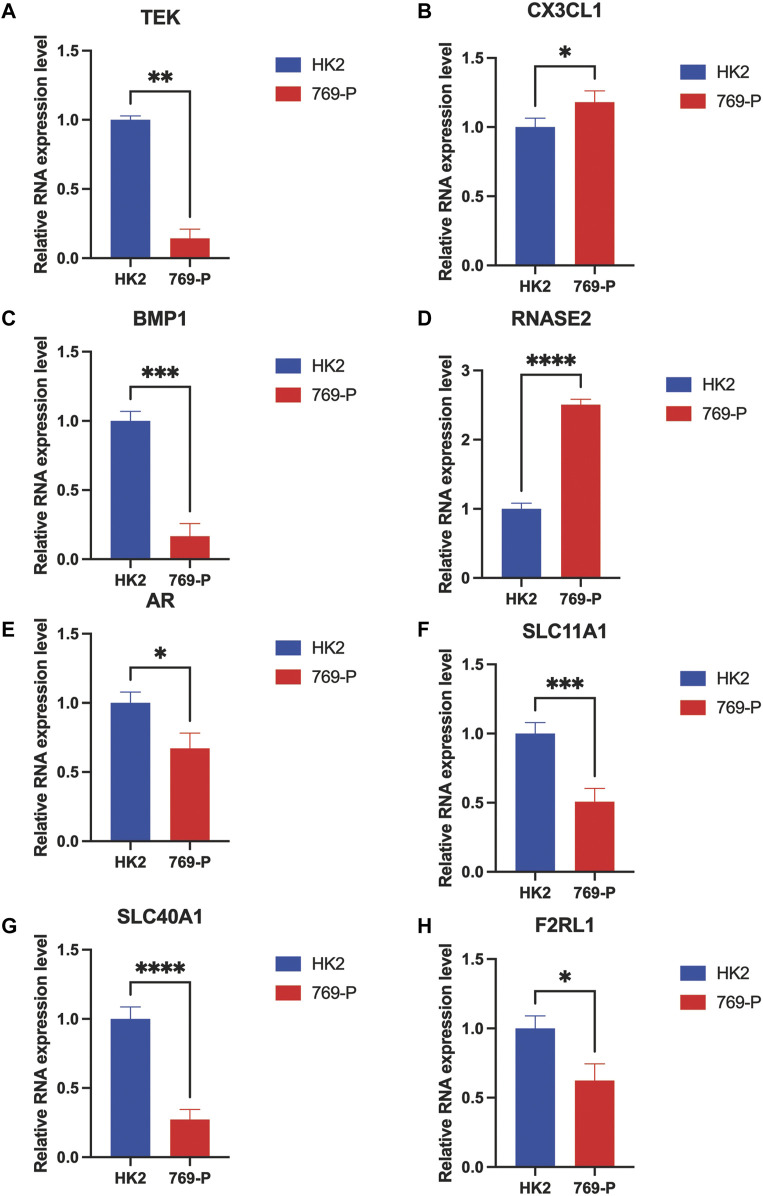
RT-qPCR confirmed the difference of the prognostic gene expression in normal renal and renal cell carcinomas. *p < 0.05, **p < 0.01, ***p < 0.001, and ****p < 0.0001.

To validate the differential expression of these immune-associated prognostic genes, RT-qPCR was employed to analyze the mRNA expression *in vitro*. The mRNA levels of TEK, BMP1, AR, SLC11A1, SLC40A1, and F2RL1 were significantly downregulated in tumor cells compared with normal cells, and CX3CL1 and RNASE2 were upregulated in tumor cells (Figure 7). However, the mechanism of action of each gene in ccRCC requires further investigation.

## Discussion

Despite targeted therapies, poor survival rates and advanced metastasis of ccRCC treatment are still challenging issues (Gu et al., 2020). The advent of immunotherapy against ccRCC, such as the targeting of PD-1, PD-L1, and CTLA4, has recently been demonstrated to revolutionize the clinical treatment for ccRCC (Farkona et al., 2016). Nevertheless, a significant number of patients still fail to respond to targeted therapy mainly due to malignant metastasis (Makhov et al., 2018). Additionally, accurate prognostic biomarkers of treatment efficacy in ccRCC are still lacking. This situation reflects the urgent need to identify potential new biomarkers for prognosis prediction and treatment targets (Xu et al., 2019b; Hu et al., 2020).

Clear cell renal cell carcinoma is one of the most immune-infiltrated tumors in the urinary system (Şenbabaoğlu et al., 2016). In reality, it is important to determine the immune function of each patient to choose the best immunotherapy strategy and predict prognosis. In recent years, some studies have established prognostic panels for ccRCC based on immune genes to predict the prognosis of ccRCC (Şenbabaoğlu et al., 2016). Immune genes appear as a valuable tool to supplement prognosis in clinical practice. In this study, ccRCCs were reclassified into two distinct ccRCC groups, the Immunity_H and Immunity_L groups, which were defined by immune characteristics, including immune-associated genes, immune checkpoint molecules, and immunotherapy. The Immunity_H group had more immune-associated gene expressions.

Additionally, the Immunity_H group was engaged in processes related to tumorigenesis and development, including Wnt signaling, focal adhesion, ECM receptor interaction, and axon guidance. Altogether, the potential associations between cancer-related processes and immune infiltration pathways in ccRCC were validated (Lai et al., 2021).

In our current work, eight genes, *TEK*, *CX3CL1*, *BMP1*, *RNASE2*, *AR*, *SLC11A1*, *SLC40A1*, and *F2RL1*, were found to be associated with poor prognosis in ccRCC based on the above methods. The prognosis model was established, and our findings indicated that the prognosis panel is a promising predictive indicator for patients with ccRCC.

Among the eight genes used to develop the IGPM-based prognosis panel, five were reported to be associated with the prognosis of ccRCC. TEK, an immune marker that promotes apoptosis by regulating the phosphorylation of AKT, can be used for risk assessment and survival prediction (Chen et al., 2021). CX3CL1, an immunoregulatory gene that is highly expressed in inflammatory ccRCC, provides a tool that enables individualized treatment of ccRCC (Wang et al., 2021). The high expression of BMP1, bone morphogenetic protein 1, indicates poor prognosis in ccRCC (Xiao et al., 2020). RNASE2, ribonuclease A Family Member 2, an RNA binding protein, indicates a prognostic value, together with other prognostic differentially expressed immune-related genes (PDEIRGs) (Wan et al., 2019; Qin et al., 2021). AR, an androgen receptor, suppresses bone metastasis of renal cancer and acts as a valuable feature in prognosis in ccRCC (Gong et al., 2021). This encourages us to further investigate the molecular mechanisms of these genes in ccRCC in future studies.

Nevertheless, there were a few limitations to our study. First, all data analyzed in this study were obtained from online databases, and further laboratory studies and clinical trial verifications are required to assess the value of this panel in ccRCC. Second, the immune gene sequence was incomplete; therefore, future studies are needed to explore whether other immune-associated genes could be used as diagnostic markers.

This study mainly focused on immune-associated genes and their association with ccRCC prognosis. This work provided a novel predictive biomarker for prognosis and clinical outcome prediction, which could help to identify an optimal prognosis prediction strategy for ccRCC and guide future immunotherapy regimens in ccRCC patients.

## Data Availability

The datasets presented in this study can be found in online repositories. The names of the repository/repositories and accession number(s) can be found in the article/Supplementary Material.
